# Supplementary material for: MetaRibo-Seq measures translation in microbiomes
Source: Nat Commun. 2020 Jun 29;11:3268. doi: 10.1038/s41467-020-17081-z (PMC7324362; doi:10.1038/s41467-020-17081-z)
Supplement: Supplementary file 10 — Supplementary Data 7 [file 41467_2020_17081_MOESM10_ESM.zip › File2/Confidence_High_Taxonomy/109723_out.krona.html]

Javascript must be enabled to view this page.

members
magnitude
magnitudeUnassigned
count
unassigned
taxon
rank

109723\_out

5

SRS051031\_contig\_number\_35470
1

superkingdom
2
2


SRS098571\_contig\_number\_60441
2
phylum
1
1224

class
1
1236

order
1
91347

1
543
family

genus
1
544

1
35703

SRS053356\_contig\_number\_13146
species

2759
1
superkingdom

kingdom
1
4751

1
451864
subkingdom

phylum
1
5204

subphylum
5302
1

155619
1

SRS023914\_contig\_number\_contig-100\_692.149290
class


SRS013687\_contig\_number\_27976
1
